# Bilateral giant inguinoscrotal hernia: A case report

**DOI:** 10.1016/j.ijscr.2021.106467

**Published:** 2021-10-02

**Authors:** Sunil Basukala, Sabina Rijal, Bishnu Deep Pathak, Rakesh Kumar Gupta, Narayan Thapa, Raveesh Mishra

**Affiliations:** a Department of Surgery, Nepalese Army Institute of Health Science (NAIHS), Kathmandu, Nepal; b Department of Surgery, BP Koirala Institute of Health Sciences (BPKIHS), Dharan, Nepal; c Department of Anaesthesiology and Critical Care Medicine, Nepalese Army Institute of Health Science (NAIHS), Kathmandu, Nepal

**Keywords:** Case report, Compartment syndrome, Inguinal hernia, Scrotum, Surgical mesh

## Abstract

**Introduction:**

Bilateral giant inguinoscrotal hernia (GIH) is rare and creates significant challenge in surgical management. The main concern of hernia reduction to abdominal cavity is development of abdominal compartment syndrome (ACS). Different approaches for prevention of ACS after surgery have been suggested.

**Case presentation:**

We report a case of 68-year-old male with bilateral inguinoscrotal hernia for 20 years reaching just below midpoint of thigh. He presented with difficulty in micturition and mobility. Preoperative investigations were normal. He underwent bilateral mesh repair without any preoperative or intraoperative adjunct measures. No significant complication occurred in postoperative period.

**Case discussion:**

Bilateral GIH is rare and the patients usually present late. GIH has been classified into three types on the basis of extension. Type I GIH can be managed with simple hernioplasty, in both unilateral and bilateral cases. Measures like resection of hernia contents and measures to enlarge intraabdominal space are warranted in type II and III GIH. Abdominal volume can be increased by utilising techniques like Pre-operative Progressive Pneumoperitoneum (PPP), injection of Botulinum toxin A (BTA) in the anterior abdominal wall, and rotation of viable tissue. The measures can be used either alone or in combination.

**Conclusion:**

Type I GIH can be treated with simple hernioplasty with safety with monitoring for features of ACS and respiratory complications postoperatively. However, additional measures like resection of hernia contents or procedures to enlarge intra-abdominal space are warranted for type II and III GIH.

## Highlights

• Surgical management of bilateral giant inguinoscrotal hernia is challenging as reduction of hernia contents to abdominal cavity may lead to development of abdominal compartment syndrome (ACS) and cardiorespiratory complications.
• We report a case of bilateral GIH managed with bilateral mesh repair with postoperative monitoring for features of ACS and cardiorespiratory complications.
• There was no significant complication in the postoperative period and no recurrence in six month follow-up period.

## 1 Introduction

Giant inguinoscrotal hernia (GIH) is defined as hernia extending below the midpoint of inner thigh of a patient in erect position [1]. It is a rare disease whose clinical presentation ranges from otherwise asymptomatic swelling, pain, urinary complains, abdomen discomfort to complications like bowel obstruction, strangulation and even end stage renal disease [2], [3], [4], [5], [6].

Standard surgical approach has not been described for the disease since large scale comparative studies have not been performed for this uncommon condition [2], [4], [7]. Possible approaches suggested are hernioplasty with forced reduction with post-operative intra-abdominal pressure monitoring or hernioplasty with resection of contents or increased intra-abdominal volume procedures [4].

In this article, we report a rare case of bilateral giant inguinal hernia extending just below midpoint of thigh that underwent open hernioplasty without any preoperative or intraoperative adjunct measures. This case report has been reported in line with the SCARE Criteria [8].

## 2 Case presentation

A 68-year-old male, known case of bilateral inguinoscrotal hernia for 20 years presented to our center with complaint of increasing difficulty in micturition for past two years. He had multiple episodes of acute urinary retention that required catheterization. The patient couldn't walk and sit properly. The swelling had become irreducible with time. He didn't have abdominal pain, distension, and vomiting. He didn't have any co-morbidities.

**Fig. 1:**
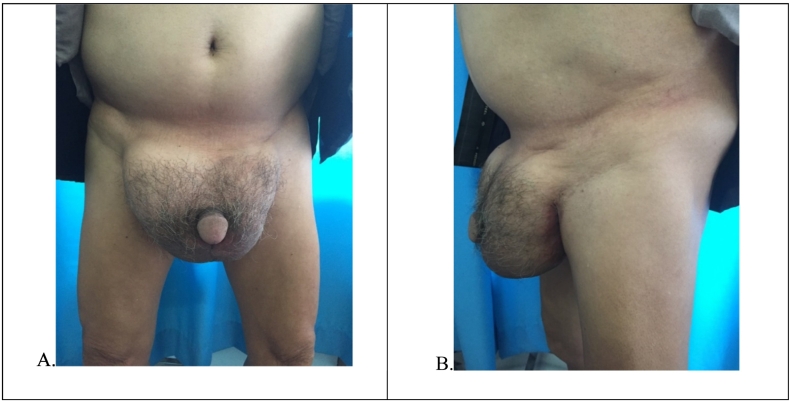
Physical examination in standing position (anterior view (A) and lateral view (B)) demonstrating bilateral giant inguinoscrotal hernia.

Physical examination revealed bilateral massive inguinoscrotal swelling that descended to just below midpoint of inner thigh in the standing position (Fig. 1). There was no evidence of inflammation, excoriation or ulceration of the scrotal skin. Penis was partially buried in detumescence state. The swelling was tender and irreducible. Cough impulse was negative and getting above the swelling was not possible. There was separate ipsilateral testicular swelling in lower one third of inguinoscrotal swelling with thickened scrotal skin and dilated veins. Haemoglobin, total leukocyte count, random blood glucose and renal function test were within normal limit.

**Fig. 2:**
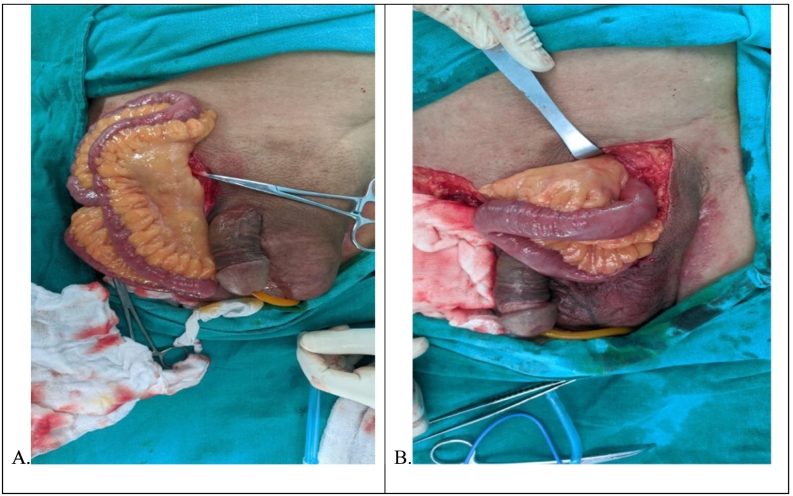
Hernia sac contents including most of the small bowel and omentum shown in right (A) and left (B) inguinal region.

Patient underwent elective exploration via inguinoscrotal approach in tertiary care hospital by a team of general and gastrointestinal surgeons and mesh hernioplasty performed on bilateral inguinal region. Intra-operatively, large hernia sac was present containing omentum and small bowel with only the proximal ileum (Fig. 2A and B). Minimal inter-bowel adhesions were found. Manual reduction of the hernia contents into the abdominal cavity was achieved through the deep inguinal ring but without any bowel resection. Adhesiolysis was done and redundant sac was excised.

**Fig. 3:**
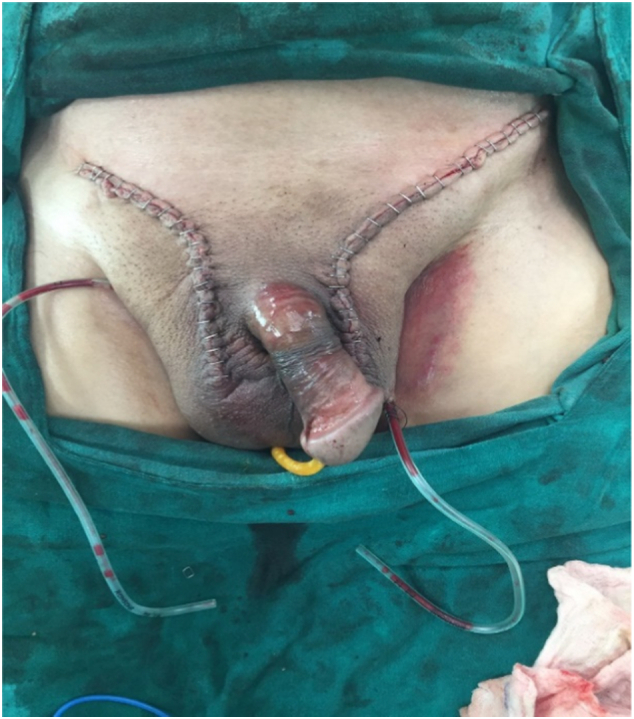
Closure of repair with Jackson Pratt drain in the scrotum.

A double-layered closure by prolene surgical mesh was done followed by reconstruction of the wall. This was followed by exploration of contralateral left inguinoscrotal hernia. Intraoperatively, we found hernia sac containing omentum and small bowel with only the proximal jejunum lying within the sac. Adhesiolysis was done and manual reduction of hernia content was done through the deep inguinal similar to left inguinal hernia. Closure was done with Prolene Mesh followed by reconstruction of wall and bilateral inguinal drain was placed (Fig. 3).

Postoperatively, there was early return of bowel movement and mobilization. His intraabdominal pressure was monitored routinely to avoid abdominal compartment syndrome. No respiratory complication was seen during his stay in hospital. On the seventh postoperative day, the drain was removed before discharge. Small postoperative inguinal seroma developed which resolved within few weeks without surgical intervention. There was no evidence of recurrence at six months during follow up.

## 3 Discussion

Giant inguinoscrotal hernia (GIH) is defined as hernia extending below the midpoint of inner thigh of a patient in erect position [1]. GIH accounts for only 2.81–5% of all inguinal hernia cases [9], [10]. With 12.5% of total GIH cases being bilateral GIHs, the occurrence of bilateral cases is even rarer [10]. Bilateral GIH occurs mostly in older adults but few cases in paediatric population have also been reported [10], [11], [12].

In 2013, Akpo conducted a study focusing on psychosocial aspects of bilateral GIH. The patients visited medical facility either due to inability of penetration or denial of sexual intercourse by partners. They presented late because of financial constraint or neglect of the disease despite understanding that surgery is necessary for treatment. These patients also experience difficulty in movement like our patient and at times face acute complications like bowel obstruction and acute retention of urine. These consequences significantly impair their quality of life. Our patient also experienced multiple episodes of acute retention of urine which can possibly be due to constrictive effect of enlarged scrotum on buried penis. In the same study, Akpo also gave classification of bilateral GIH for the first time [10]. However, any difference in principle of management as per the classification has not been suggested.

Considering the rarity of the disease and unavailability of standard approach, dilemma exists among healthcare providers for management of GIH [2], [4], [7]. The concerns of surgery in GIH are rise in intra-abdominal pressure as well as cardiorespiratory complications [3].

Trakarnsagna A et al. classified GIH into three types on the basis of extent of hernia sac, based on which management principles differ. In type I GIH (hernia that extends between midpoint of inner thigh and the midpoint of the mid-inner thigh and suprapatellar margin), hernioplasty with forced reduction with post-operative intra-abdominal and intra-thoracic pressure monitoring is considered enough. However, in other types, resection of contents or increased intra-abdominal volume procedures is warranted to prevent intra-abdominal hypertension [4]. Our patient with type I GIH underwent hernioplasty without any adjunctive pre-operative or intra-operative procedures meant for preventing intra-abdominal hypertension and still, didn't experience post-operative Abdominal Compartment Syndrome (ACS) which suggests that the principle provided by Trakarnsagna A et al. holds true even for bilateral GIH.

One study conducted among 32 patients with type I GIH including a case of bilateral GIH showed that laparoscopic surgery using Transabdominal Pre-peritoneal (TAPP) approach with negative pressure drain can be performed with safety and low post-operative complications [5]. This study also supports the principle of management given by Trakarnsagna et al. in bilateral cases. The additional advantages of TAPP among patients with type I GIH in comparison to the open hernioplasty that was performed in our patient include smaller wound size, eligibility of procedure in patients with skin infections, easier reduction of contents and coverage of whole defect by mesh with convenience [5], [13]. Concern regarding incisional or mesh infection due to large incision in open repair have been raised [13]. However, with proper care of wound, such complication wasn't noted in our case.

In type II and type III GIH with loss of domain, additional procedures to prevent ACS should be considered. Various approaches used for prevention are resection of hernia contents and procedures to enlarge intra-abdominal volume [4]. Abdominal volume can be increased by utilising techniques like Pre-operative Progressive Pneumoperitoneum (PPP), injection of Botulinum toxin A (BTA) in the anterior abdominal wall and rotation of viable tissue (Example: component separation technique) [4], [13].

The techniques mentioned earlier are either used alone or in combination for GIH (including bilateral GIH) as mentioned in some recent studies [4], [13], [14]. A patient of 65 years with bilateral GIH that extended to a plane just above knee was treated using simultaneous PPP and injection of botulinum toxin followed by hernioplasty using Stoppa technique. His recovery was uneventful with no complication during the fifteen month follow-up period [14]. In a study of eight patients including two cases of bilateral GIH using similar combination for preoperative preparation followed by herniopalsty using TAPP approach was studied. The post-operative period was uneventful with no occurrence of ACS or respiratory complication in any patient. There was no recurrence during the follow-up period of 9–20 months [13].

Despite emergency surgical intervention, a rare case of fatality in bilateral GIH has been reported in an obese patient presenting with respiratory distress [15]. This case provides insight into the gravity that the condition can lead to in selected patients.

## 4 Conclusion

Type I GIH, including bilateral GIH, can be treated safely with simple hernioplasty. However, patient should be monitored vigilantly for features of ACS and respiratory complications during the postoperative period. In type II and type III GIH, additional measures like resection of hernia contents or procedures to enlarge intra-abdominal space are required.

## Ethical approval

The case report is exempt from ethical approval in our institution.

## Funding

This research did not receive any specific grant from funding agencies in the public, commercial, or not-for-profit sectors.

## CRediT authorship contribution statement

Sunil Basukala (SB), Sabina Rijal (SR), Narayan Thapa (NT), Rakesh Kumar Gupta (RKG) = Study concept, Data collection, and surgical therapy for the patient

SB, SR, Bishnu Deep Pathak (BDP) = Writing - original draft preparation

RM, SR, BDP = Editing and writing

NT and RKG = senior author and manuscript reviewer.

All the authors read and approved the final manuscript.

## Guarantor

Sunil Basukala.

## Research registration number

Not applicable.

## Consent

Written informed consent was obtained from the patient for publication of this case report and accompanying images. A copy of the written consent is available for review by the Editor-in-Chief of this journal on request.

## Provenance and peer review

Not commissioned, externally peer-reviewed.

## Declaration of competing interest

None.
